# Not Guilty on Every Count: The “Non‐Innocent” Nitrosyl Ligand in the Framework of IUPAC′s Oxidation‐State Formalism

**DOI:** 10.1002/anie.202003122

**Published:** 2020-05-26

**Authors:** Torsten Ampßler, Georg Monsch, Jens Popp, Tobias Riggenmann, Pedro Salvador, Daniel Schröder, Peter Klüfers

**Affiliations:** ^1^ Department Chemie der Ludwigs- Maximilians-Universität Butenandtstraße 5–13 81377 München Germany; ^2^ Institut de Química Computacional i Catàlisi i Departament de Química Universitat de Girona Maria Aurèlia Capmany 69 17003 Girona Spain

**Keywords:** cobalt, coordination chemistry, nitrosyl ligands, oxidation state, vanadium

## Abstract

Nitrosyl–metal bonding relies on the two interactions between the pair of N–O‐π* and two of the metal's d orbitals. These (back)bonds are largely covalent, which makes their allocation in the course of an oxidation‐state determination ambiguous. However, apart from M‐N‐O‐angle or net‐charge considerations, IUPAC′s “ionic approximation” is a useful tool to reliably classify nitrosyl metal complexes in an orbital‐centered approach.

## Graphical abstract

**“The MNO segment should be linear for NO** ^**+**^ **but bent for NO** ^**−**^ **”**: Though this statement is taken from a IUPAC publication on oxidation states (OS), it mirrors a common belief. However, deriving the OS by the effective‐oxidation‐state procedure (EOS), the new nitrosylcobalt species (see the Figure) resembled a Co^I^(NO^+^) couple instead of the expected Co^III^(NO^−^).

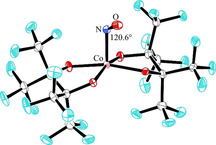

The nitrosyl ligand is *the* prototypic example of a so‐called “non‐innocent” ligand—a subject that was dealt with by Jørgensen, who attributed innocence to a ligand if it allowed the unambiguous assignment of the central metal's oxidation state (OS).^1^ The [Fe(H_2_O)_6_]^2+^ ion is a typical example thereof, allowing a clear‐cut assignment of the iron atom's OS, even without the need for a tightly fixed OS definition. The replacement of one, innocent, aqua ligand with nitric oxide results in the [Fe(H_2_O)_5_(NO)]^2+^ ion, in which the iron atom's OS becomes a matter for discussion.^2^ At this point, a clear concept of what the OS is, becomes the prerequisite to enter a debate. The IUPAC in 2016 provided a recommendation, accompanied by two clarifying documents.^3^ There, the OS is the charge of an atom or a molecular fragment after the ionic approximation (IA) of its heteronuclear bonds. The IA may be extracted from the mixing coefficients of molecular orbitals by assigning the electron pair in question to the atom with the major contribution to the bond and the minor to the antibond.^4^

In the case of a nitrosyl–metal fragment, the IA has to be performed on three interactions: one formal M←NO^+^ donor bond of virtually pure ligand character which goes to NO on IA, and two M–(NO‐π*) bonds, the character of which may vary between an M→NO^+^ backbond and an M←NO^−^ donor‐bond scenario.^5^ These latter two interactions are the origin of all the ambiguities within the nitrosyl metal field.

In fact, IUPAC′s IA applied to (canonical, natural or localized) MOs is actually fit to master both real and pretended ambiguities. An obviously indestructible example of the latter deals with the M‐N‐O angle: is it correlated to the NO ligand's charge—or does it actually indicate the charge? The IUPAC statement is clear: “the MNO segment should be linear for NO^+^ but bent for NO^−^”.3a

Missing a consistent guideline, the nitrosyl metal community has continued to apply workarounds for the assignment of OSs such as taking the nearest integers of net charges obtained from population analyses.^6^ In the two‐bond scenario of a metal nitrosyl, however, net charges and the IA‐derived OSs may actually come out with different signs, as will be shown below. In order to 1) demonstrate the benefit of the basic IA procedure in the area of nitrosyl metal species, 2) improve the inadequate IUPAC treatment of the nitrosyl‐related examples, and 3) deal with the relationship of net charges and OSs in the nitrosyl metal field, we present three isostructural nitrosylmetallates which formed as adducts of nitric oxide to a special class of low‐coordinate bis(diolato)metallates, the tetracoordinate bis(perfluoropinacolato)metallates [M(fpin)_2_]^2−^ of chromium(II), iron(II), and cobalt(II). The chemistry of the latter two species has attracted interest due to the low energetic cost of planarizing them to high‐spin, square‐planar coordination entities which, moreover, have only a limited tendency to bind additional donor ligands.^7^ The new high‐spin, square‐planar chromium(II) complex of this work complements the series (see Supporting Information).

All three precursors add nitric oxide to form bis(diolato)nitrosylmetallates (Scheme 1). All reactions proceeded through the coupling of the NO's unpaired spin to one of the spins of the high‐spin metal centers (*S=*2 for d^4^‐Cr^II^ and d^6^‐Fe^II^, *S=*3/2 for d^7^‐Co^II^). In addition to the spin coupling on M–NO bond formation, the cobalt and the chromium centers experienced a high‐to‐low‐spin transition on NO‐coordination. After crystallization, we obtained the diamagnetic (NMe_3_Bn)_2_[Co(fpin)_2_(NO)] (**1**), the spin‐1/2 compound (NHEt_3_)_2_[Cr(fpin)_2_(NO)] (**2**), and the spin‐3/2 compound (NHEt_3_)_2_[Fe(fpin)_2_(NO)] (**3**). In terms of the Enemark–Feltham notation we are dealing with singlet‐{CoNO}^8^, doublet‐{CrNO}^5^, and quartet‐{FeNO}^7^ species. In addition, a salt of the known singlet‐{VNO}^4^ monoanion, Na_2_[V(NO)(tea)]I⋅5 H_2_O (**4**, tea=triply deprotonated triethanolamine), was included.^8^

**Scheme 1:**
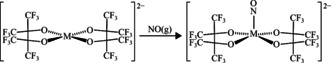
The reaction of tetracoordinate perfluoropinacolatometallates with nitric oxide (M=Cr, Fe, Co) to yield **1**–**3**.

We start our survey with the cobalt compound. Most {CoNO}^8^ compounds exhibit a square‐pyramidal (*SPY*‐5) structure that features an angulate Co‐N‐O link. In a biochemical context, the addition of NO to cobalamines(II) produces members of this class.^9^ Less frequently, trigonal‐bipyramidal (*TBPY*‐5) variants with a linearly bonded, equatorial nitrosyl ligand have been reported; both forms have been found to coexist for chlorido/phosphane co‐ligands where the mapping of the bent/linear CoNO fragments to NO^−^/NO^+^ and to Co^III^/Co^I^ led to the formulation of valence tautomerism.^10^

When a solution of the precursor [Co(fpin)_2_]^2−^ in methanol was submitted to an NO atmosphere under in‐situ‐IR control, a mononitrosyl complex formed immediately which, after a delay of about an hour, was transformed into the dinitrosylcobaltate ion [Co(fpin)(NO)_2_]^−^.^11^ If the NMe_3_Bn^+^ counterion was still present on NO supply, rapid crystallization within the available hour led to reddish brown crystals of **1**. The anion of **1** was rather unstable. On a purge of inert gas, both the solution and the crystals decomposed rapidly under NO liberation.

Figure 1 shows the *SPY*‐5 coordination of the nitrosylcobaltate with the typical tilt of the NO ligand. The electronic structure was evaluated by means of DFT and CASSCF calculations which confirmed the singlet state. As described for {FeNO}^7^ compounds, the Co−NO bonds are weakened in the sense of static correlation (see the bond/antibond population in Figure 2), an interpretation of which, in terms of enhanced Pauli repulsion, is given in Ref. 2. Figure 2 shows the frontier MOs from a CASSCF(8,7) calculation (the seven MOs of the entire active space are shown in the Supporting Information). On visual inspection, the Co−NO π‐bond (MO 181) appears to be cobalt‐centered and, thus, resembles a π‐backbond. An ambiguity is raised by the Co−NO σ‐bond (MO 182). Examined together with its antibond (MO 183), the almost perfect covalency (in terms of comparable contributions of Co and NO to bond and antibond) becomes obvious—a frequent case in nitrosylcobalt chemistry as well as in nitrosyl metal chemistry in general.^12^ In order to perform the IA reliably, a validated method instead of the mere inspection of MOs was required. For this task, the effective‐oxidation‐state (EOS) method was applied.^13^

**Figure 1:**
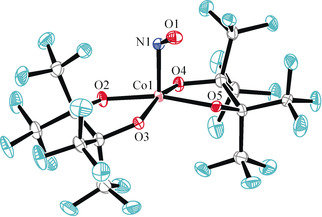
Ortep plot of the major disorder form (81 %) of the anion of **1**, drawn at 70 % ellipsoid probability. Distances in Å and angles in °: Co to: N1 1.793(2), O2 1.899(2), O3 1.904(3), O4 1.898(3), O5 1.901(2); N–O 1.185(3), Co‐N‐O 120.6(2).

**Figure 2:**
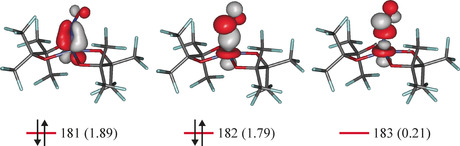
The frontier orbitals 181–183 of the anion of **1** in a CASSCF(8,7) approach (isovalue 0.06 a.u.); the population is given in parentheses, the arrows represent the ground state's leading 2222000 configuration (79 % contribution).

As a result, all eight electrons (in MOs 179–182, see Supporting Information) of the {CoNO}^8^ fragment fall to cobalt, which, then, is a d^8^ cobalt(I) center acting as a double donor towards an NO^+^ electrophile (the π‐backbond MO 181 and the “σ‐backbond” MO 182 in Figure 2). It should be repeated at this point that the OS is drafted as a winner‐take‐all principle. Hence, other members of the {CoNO}^8^ family with a slight shift of the σ‐bond's electron density towards the ligand may end up as a Co^III^(NO^−^) case. This is neither a weakness of the procedure nor of the concept, it is simply the trace of covalence—and it has little to do with the 120° Co‐N‐O angle. That statement is illustrated by a scan for metastable configurations of the anion of **1** as a preliminary for the search of photoinduced linkage isomers (PLIs).^14^ Among the candidate PLIs, some 80 kJ mol^−1^ above the *SPY*‐5 ground state, we found the analogue of the above‐mentioned *TBPY*‐5 isomer (**1′**) with a linearly bonded NO ligand in the bipyramid's equatorial plane (see the Supporting Information). As a result of the EOS analysis, the same OSs are assigned to both isomers. Hence, an NO^+^ ligand has two choices when faced with a low‐spin d^8^‐metal center: first, as found in **1**, the metal‐d${}_{z{}^{2}}$ pair binds laterally into one lobe of the N–O‐π* MO (MO 182 in Figure 2) complemented by a Co–NO‐π‐interaction (MO 181). Second, as in **1′**, the NO ligand may rotate into Co‐N‐O linearity and replace the Co−NO σ‐bond by a second π‐bond using the d_*xz*_ donor pair. In conclusion, the two bonding modes resemble linkage isomers, rather than valence tautomers.

Do electronegativity (*χ*) arguments contribute to these questions? The IUPAC based the applicability of *χ*‐tables on the Haaland criteria (normal bonds, both covalent and ionic, dissociate homolytically, dative bonds dissociate heterolytically):^15^ “If the split is heterolytic, the ionic approximation follows the electrons; if homolytic, electronegativity applies.”3a For the {CoNO}^8^ species **1**, the thermal split of the Co–NO interaction is the reverse of the species formation [Eq. (1)]:

(1) $$d{}^{7}\text{-}\mathrm{Co}{}^{\mathrm{II}}+\mathrm{NO}{}^{•}\vec{\leftarrow }\left\{\mathrm{CoNO}\right\}{}^{8}$$

According to the Haaland formalism, the Co–NO interaction, thus, is established by one normal bond, say the σ‐bond, and one dative bond, say the π‐backbond. (The one electron spent for the normal bond corresponds to the historic way of counting NO in a bent link as a one‐electron donor.5b) The flow‐back of the π‐bond's two electrons to cobalt both on real bond cleavage and on OS determination mirrors its nature as a backbond. The allocation of the homolytically cleaving normal Co−NO σ‐bond to cobalt on OS determination, however, might be unexpected for those who assume *χ*_NO_ close to *χ*_N_ and *χ*_O_. In fact, an NO^+^ ion has a much lower‐than‐expected tendency to accumulate additional charge in its π*‐orbitals as is demonstrated by a simple salt: a polar, but largely covalent ON−OSO_3_H bond might be contemplated in nitrosyl hydrogensulfate due to *χ*_O_‐*χ*_N_ of 0.55 on the Allen scale. It is, in fact, ionic, modifying the IUPAC statement to “… if homolytic, *group* electronegativity applies”.^16^

At this point a look at the relationship between the OSs and net charges, N−O bond orders and related measures is helpful. To start with the latter, Figure 3 (left), in terms of a Badger's‐rule plot, shows that the compounds compiled here behave regularly with respect to bond‐order‐related parameters such as the N–O stretch and the N–O distance.^17^ Figure 3 (right) then focuses directly on a net charge, the QTAIM charge. How is a net charge related to the OS? Since the Co−NO σ‐bond is close to ideal covalency, the net transfer of charge resembles a little less than one electron to NO^+^ through the σ‐bond, increased by a Δ*q* clearly lower than one from the π‐backbond. Starting from NO^+^, the net charge on the nitrosyl ligand is, thus, about −Δ*q* due to the charge flow through the two M→NO bonds—in terms of the QTAIM charge about −0.35 *e*.

**Figure 3:**
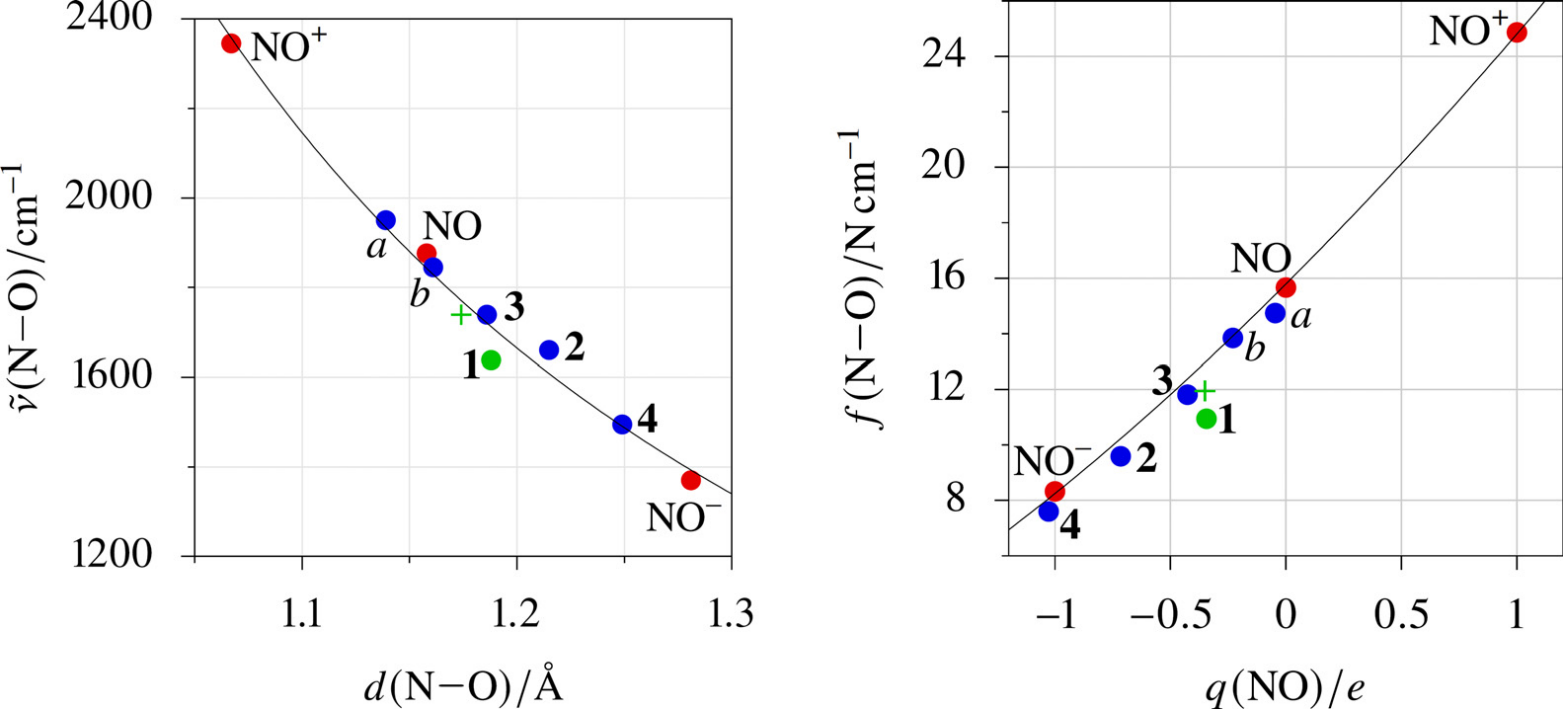
Left: experimental wavenumbers $\tilde{\nu }$ of the N–O stretch of solid samples of **1**–**4** as a function of the N–O distance *d*. The reference line is a fit according to Badger's rule applied on the free NO^+/0/−^ species. Included are two known iron species: *a* [Fe(CN)_5_(NO)]^2−^, *b* [Fe(H_2_O)_5_(NO)]^2+^. The “**+”** marks the calculated $\tilde{\nu }$ of **1′**. Right: force constants *f* of the N−O bonds as a function of the NO's QTAIM charge *q* for **1**–**4**, **1′**, *a*, *b*.

In view of the negative QTAIM charge of the NO ligand in **1**, an NO^+^‐assignment, at first glance, might feel counterintuitive. However, focusing on the two dominant bonds that fill the N–O‐π* MOs, their electron pairs would fall to NO^+^ upon OS assignment not before the nitrosyl's share exceeds one electron per bond. In the special case of two equivalent acceptor bonds in a linear M‐N‐O moiety of *C*_3v_ or *C*_4v_ symmetry, we would end up with NO^+^ until a net NO charge near −1 is reached, and would then, by the allocation of two electron pairs at the ligand, switch from NO^+^ to NO^3−^. Notably, *the* prototypic NO^+^‐type nitrosyl metal complex, nitroprusside, [Fe(CN)_5_(NO)]^2−^ (species *a* in Figure 3), is far from this limit with its net charge close to zero.

When the literature is browsed for species with borderline behavior with respect to any of the coordinates of Figure 3, Wieghardt's diamagnetic [V(NO)(tea)]^−^ (**4**) ion comes into focus (see Figure 4 and the Supporting Information for the parameters from a re‐determination using the synthetic procedure of Ref. 8b).8a Visual inspection of the two equivalent V–NO‐π‐bonds and their antibonds (see Supporting Information) show, again, their largely covalent nature. In the EOS analysis, however, both electron pairs go to the ligand. As a result, we end up with an NO^3−^ ligand and a vanadium(V) central atom (Wieghardt assigned a V^I^/NO^+^ couple due to the linearity of the V‐N‐O link and formulated a “reductive nitrosylation”). However, this example nicely shows the usefulness of the OS formalism: Wieghardt prepared the ion, according to Griffith et al., by the reaction of vanadate(V) and hydroxylamine, “H_3_(NO)”, in alkaline aqueous solution.^18^ In terms of OSs, this is not a redox reaction at all but the threefold deprotonation of hydroxylamine and the bonding of its triply conjugate base to the still pentavalent central metal.

**Figure 4:**
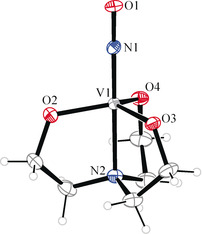
Ortep plot of Wieghardt's anion in **4**, 70 % ellipsoid probability, the ethylene bridges of the tea ligand are drawn for the major disorder form (85 %). Distances in Å and angles in °: from V1 to: N1 1.697(2), N2 2.167(2), O2 1.896(1), O3 1.890(1), O4 1.893(1); N1–O1 1.253(2); V1‐N1‐O1 177.9(2).

Including paramagnetic species broadens the discussion. In analogy to the preparation of **1**, the chromium compound **2** was obtained as violet crystals from a solution of the square‐planar precursor complex (NHEt_3_)_2_[Cr^II^(fpin)_2_] on NO exposure. Unlike **1**, both the solution and the crystals of **2** are of distinctly higher stability. Structure analysis showed a linear Cr‐N‐O moiety with an N–O distance intermediate between **1** and **4**, a short Cr−N bond, a rather low $\tilde{\nu }$ for the N–O stretch and a rather negative charge on NO (Figures 3 and 5). Inspection of the MOs from a CASSCF(5,7) calculation hints at rather metal‐centered Cr−NO π‐bonds, which, thus, appear to be π‐backbonds.

**Figure 5:**
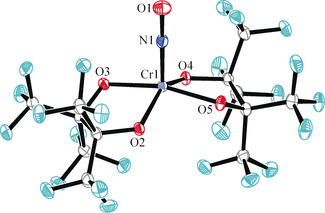
Ortep plot of the anion of **2**, drawn at 50 % ellipsoid probability. Distances in Å and angles in °: from Cr1 to: N1 1.655(2), O2 1.922(2), O3 1.992(2), O4 1.917(2), O5 1.982(2); N–O 1.214(2), Cr‐N‐O 179.5(2).

However, EOS analysis resulted in a Cr^III^/NO^−^ instead of a Cr^I^/NO^+^ couple. The reason for the NO^−^ assignment is the spin polarization within the two bonds which is driven by the single excess spin in the d_*xy*_ orbital of this *S=*1/2 species. As illustrated in the Supporting Information, we see the Cr−NO π‐bonds orthogonal to the α‐spin in d_*xy*_. The α‐spins of the π‐bonds, thus, behave “Hund's‐rule‐like” by being concentrated closer to the central metal's α‐spin, leaving the β‐counterparts closer to the NO ligand. Again, the Cr–NO interaction is largely covalent, but now, in the EOS procedure the α‐part of each bond pair falls to the metal, and the β‐part to the ligand. In total, the IA of the individual spins allocates both β‐spins of the two π‐bonds to NO^+^ which, thus, turns into NO^−^. Keeping in mind that spin polarization of electron pairs on orthogonal interaction with singly occupied metal‐d orbitals is a frequent scenario in the coordination chemistry of the 3d‐transition metals, a broadening of the OS definition may be sensible. In fact, all other published procedures to derive OSs from the wavefunction rely on α/β‐separation as well.^19^

This aspect is underlined by the final example of this report, the reddish brown iron compound **3**. Addition of NO to the square‐planar precursor (see Supporting Information) yielded a member of the quartet‐{FeNO}^7^ subclass of nitrosyliron species. Figure 6 shows the anion's structure. With its O‐only coordination in the co‐ligand part, **3** is related to the [Fe(H_2_O)_5_(NO)]^2+^ parent aqua species of this class.^2^ Accordingly, **3** shares key properties with the aqua ion such as the repulsion of the nitrosyl ligand's N‐centered lone pair and the singly populated Fe‐d${}_{z{}^{2}}$ orbital which makes the Fe−N bond about 0.1 Å longer than the Cr–N distance in **2**, and gives the nitrosyl ligand its typical tilt. However, despite the close similarity between **3** and the parent aqua species, the EOS analysis revealed an NO^−^ ligand in **3** (due to the allocation of two β‐spins at the ligand), but NO^+^ for the aqua species.^2^

**Figure 6:**
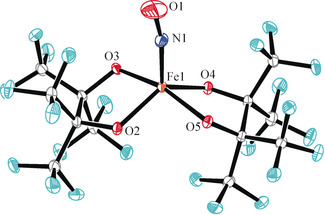
Ortep plot of the anion of **3**, 50 % ellipsoid probability. Distances in Å and angles in °: from Fe1 to: N1 1.757(2), O2 1.943(1), O3 2.042(1), O4 1.953(1), O5 2.058(1); N–O 1.149(2); Fe‐N‐O 168.5(2).

As an overview of the diverse results of the IA procedures herein, Scheme 2 gives a sketch of the electron allocations in the winner‐take‐all scenario of an OS assignment. In particular, the quartet‐{FeNO}^7^ species show a distinct α/β spread which is driven by three excess spins with the result that only the minority spin is found in covalent interaction—be it ligand‐ or metal‐centered—with the majority spin at the metal (“β‐covalence”).

**Scheme 2:**
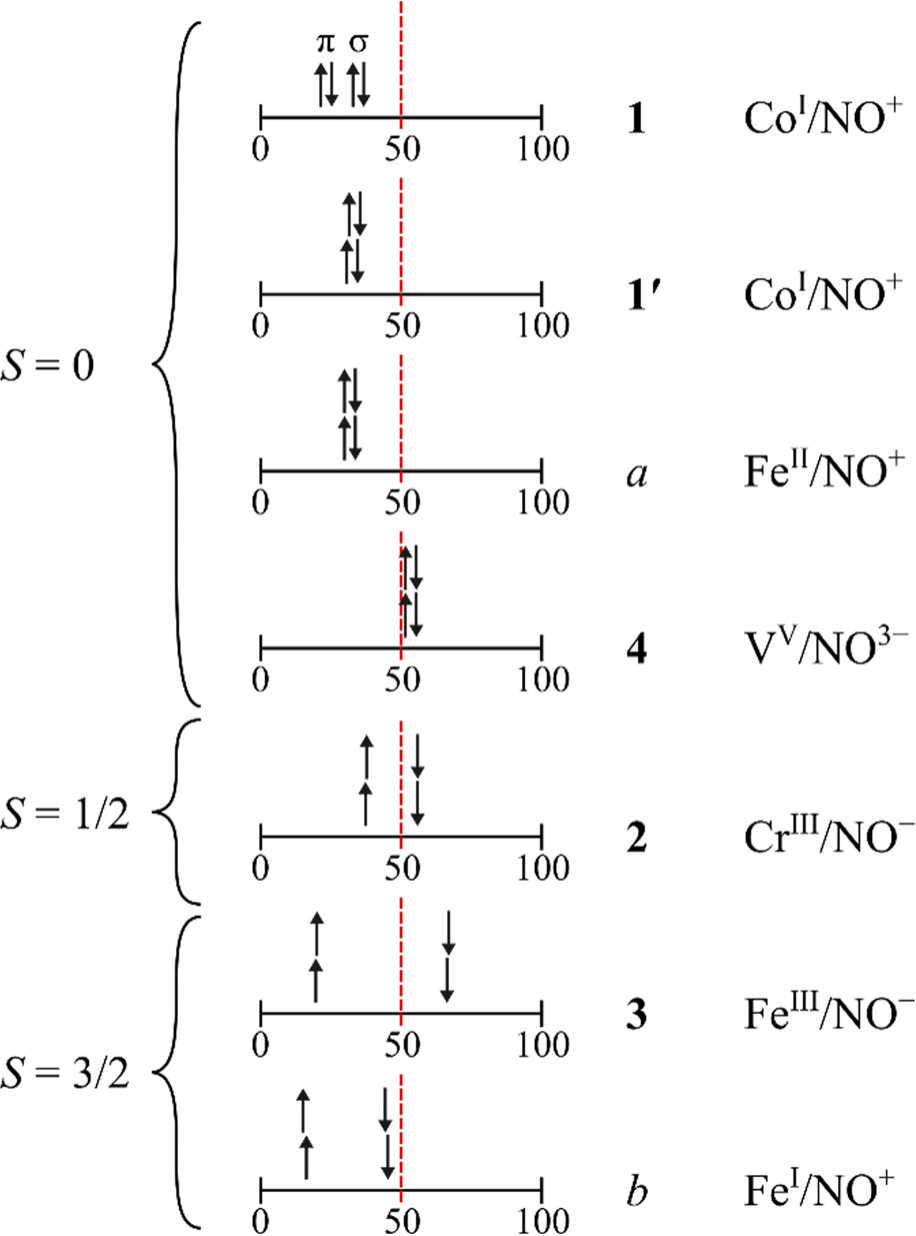
A sketch of the various scenarios to find an electron right of the M–NO bond's 50 %‐NO‐character threshold, thus falling to NO on IA. For *S=*0, the double arrows denote a non‐spin‐polarized pair. Except for **1**, all electron pairs are part of M–NO‐π‐bonds.

In conclusion, the nitrosyl ligand's “non‐innocence” stems from a property of the two occupied M–NO bonds of the frontier‐orbital region, which always imposes a problem in the OS framework: they are largely covalent. Thus, small shifts in the actual charge distribution within a bond close to the 50 % threshold make the OSs jump to up to four units. Figure 7 shows this fact for the two extremes examined in this work: the clear NO^+^ case of nitroprusside (right) and the borderline NO^3−^ case of Wieghardt's vanadate (left). Both species are singlets, and have degenerate bond pairs. Despite the fact that we merely see covalent π‐bonds in both cases, with a bit more NO character in the vanadate and a bit more metal character in the ferrate, the OSs experience a four‐electron leap—which maps onto a one‐unit‐charge step for the net charges (and, by the way, no change of the M‐N‐O angle).

**Figure 7:**
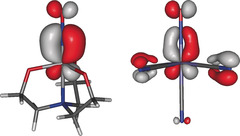
One of the two degenerate M‐NO π‐bonds of **4** (left) and *a* (right). MOs from CASSCF(4,4) for **4**, CASSCF(6,7) for *a*; isovalue in a.u.: 0.06.

Spin polarization in non‐singlet species seems to bedevil the situation further. However, when asking for the origin of the marked extent of spin polarization in nitrosyls—which is driven by the metal, not the ligand—the answer recurs: again, it is the covalency of the two decisive M–NO (back)bonds that fosters spin communication.

To get back to the title statement: nitrosyl ligands are non‐innocent. However, it's only covalency, the common antagonist of OS considerations also in the field of ligands usually classified innocent.^20^

## Conflict of interest

The authors declare no conflict of interest.

## Supporting information

As a service to our authors and readers, this journal provides supporting information supplied by the authors. Such materials are peer reviewed and may be re‐organized for online delivery, but are not copy‐edited or typeset. Technical support issues arising from supporting information (other than missing files) should be addressed to the authors.

Supplementary
