# Supplementary material for: Not Guilty on Every Count: The “Non‐Innocent” Nitrosyl Ligand in the Framework of IUPAC′s Oxidation‐State Formalism
Source: Angew Chem Int Ed Engl. 2020 May 26;59(30):12381–6. doi: 10.1002/anie.202003122 (PMC7384168; doi:10.1002/anie.202003122)
Supplement: Supplementary file 1 — Supplementary [file ANIE-59-12381-s001.pdf]

Angewandte Chemie
Eine Zeitschrift der Gesellschaft Deutscher Chemiker

Supporting Information

Not Guilty on Every Count: The "Non-Innocent" Nitrosyl Ligand in the Framework of IUPAC′s Oxidation-State Formalism

*Torsten Ampßler, Georg Monsch, Jens Popp, Tobias Riggenmann, Pedro Salvador, Daniel Schröder, and Peter Klüfers**

anie_202003122_sm_miscellaneous_information.pdf

Supplementary Information

Contribution of the Munich group

Preparation of crystals of 1 – 4

1 – **3** were prepared by the submission of a methanolic solution of the in-situ-prepared precursors $(NMe_3Bn)_2[Co(fpin)_2]$, $(NHEt_3)_2[Cr(fpin)_2]$, and $(NMe_3Bn)_2[Fe(fpin)_2(NO)]$ to a nitric-oxide atmosphere. The three compounds are tetracoordinate metallates with square-planar (Cr, Fe) coordination, or a coordination intermediate between square-planar and tetrahedral (Co). Their preparation, X-ray structures, UV-vis spectra and computational analysis will be published elsewhere.

Crystals of **2** were prepared as follows: perfluoropinacol (36.0 µL, 200 µmol, 2 eq.) was added to a methanolic solution of chromium(II) chloride (3.00 mL, 0.033 M, 100 µmol, 1 eq.). Upon treatment with triethylamine (56.0 µL, 400 µmol, 4 eq.), the light blue solution first turned blue and afterwards a greyish suspension was formed. The argon atmosphere was replaced by gaseous nitric oxide leading to a dark violet solution. Violet crystals were obtained within a few minutes.

Crystals of **3** were prepared as follows: perfluoropinacol (333 mg, 178 µL, 968 µmol) was added to a solution of iron(II) triflate (171 mg, 484 µmol) in methanol (24 mL). After treatment with triethylamine (270 µL, 1.93 mmol) the solution turned blue, which was accompanied by the formation of a lavender precipitate. The suspension was vigorously stirred in an atmosphere of nitric oxide, leading to a color change of both solution and precipitate to burgundy. The nitric-oxide atmosphere was removed and water (24 mL) was added to the reaction mixture. After filtration, the product was washed with water (3×5 mL), dried *in vacuo*, and recrystallised from methanol.

In a modification of the procedure in Ref. [1], crystals of **4** were prepared from a solution of hydroxylamine hydrochloride (42.0 mg, 0.60 mmol) and NaOH (60.0 mg, 1.50 mmol) in 0.5 mL water which was added to a suspension of NH_4VO_3 (35.0 mg, 0.30 mmol) and triethanolamine (40 µL, 0.30 mmol) in 0.5 mL water. The mixture turned to dark red and cleared up immediately. Sodium iodide (1.50 g, 10.0 mmol) was added. A red solid formed at 2 °C within two days. After washing with diethyl ether (3×20.0 mL) it was dried *in vacuo* and re-dissolved in ethanol to a saturated solution. Pink crystals of **4** grew on diffusion of dimethyl sulfoxide vapours into the solution at room temperature within two days.

X-ray analyses

Details of the crystallographic characterisation of **1** – **4**, including the CCDC numbers, are collected in two tables at the end of the SI.

Spectroscopic characterisation

IR wavenumbers are given below in the comment to Figure 3. UV-vis spectra of all compounds as well as NMR spectra of the diamagnetic vanadate will be published elsewhere.

Computations

Structure optimisation and numerical frequency analyses on the DFT level were performed by Orca 4.2.1.[2]

Computational details for the cobaltate in 1

The recently published Ref. [4] deals with $\{CoNO\}^8$ species and their reduced forms. There, the ground state of a diamagnetic *SPY*-5 $\{CoNO\}^8$ complex was described as a singlet-biradical with the true singlet 6.02 kcal mol^{-1} above the biradical (Table 1 of the reference).

For **1**, this finding was checked in terms of a broken-symmetry approach. As a result, we found dependence on the method. With the GGA functional BP86 the BS approach fell back to the singlet, whereas the hybrid functional B3LYP ended up with a singlet-biradical ground state, some 6 kJ mol^{-1} more stable than the true singlet and an overlap of the corresponding MOs of $S_{\alpha\beta} = 0.81$.

The *TBPY*-5 isomer **1’** is a local minimum on the singlet’s potential energy surface, ca. 80 kJ mol^{-1} above the *SPY*-5 ground state in terms of a B97-D3-ZORA/def2-TZVP+CPCM(∞) calculation. Figure S1 shows the molecular structure. The wavenumber of the N–O stretch is 1739 cm^{-1}.

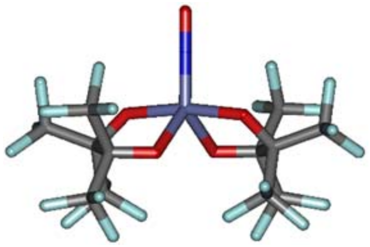

Figure S1. The metastable, C_2-symmetric *TBPY*-5 linkage isomer **1’** of the $[Co(fpin)_2(NO)]^{2-}$ ion. Distances in Å and angles in °: Co to: N1 1.635, O_{ax} 1.922, O_{eq} 1.989; N–O 1.174, Co–N–O 180.0.

Figure S2 shows the frontier orbitals from CASSCF(8,7)/def2-TZVP+CPCM(∞) calculations on **1** and **1’**.

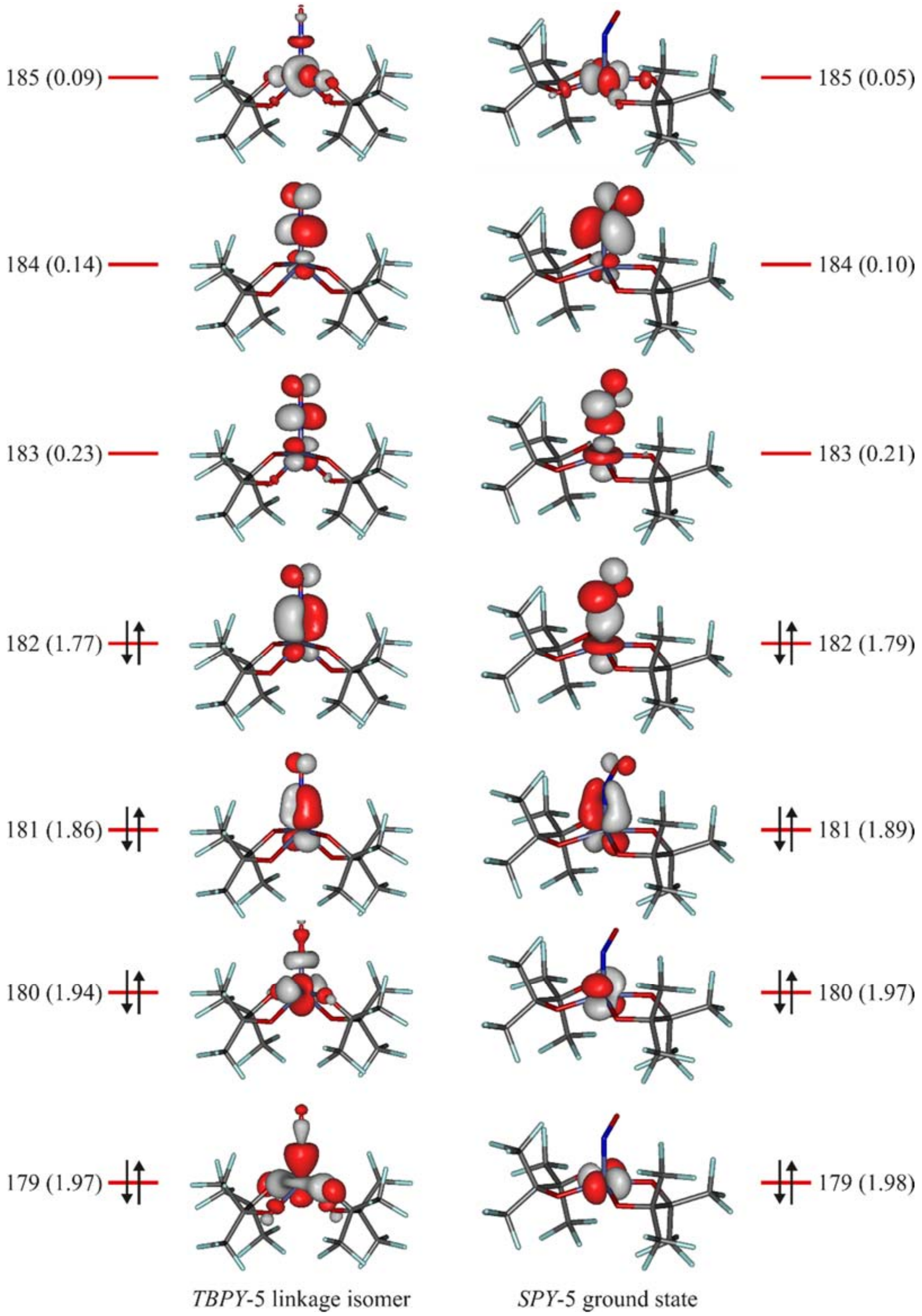

Figure S2. The MOs of the active space of CASSCF(8,7) calculations on **1** (right) and **1'** (left), isovalue 0.06 a.u.; the population is given in parentheses, the arrows represent the ground state's leading 2222000 configuration (79 % contribution for both species). Note the higher antibond population for the π-bonds. Static correlation is larger for π-bonds due to the generally lower overlap compared to σ-bonds.

Details for Figure 3

Left: experimental $\tilde{\nu}$ values of the N–O stretch of solid samples of **1**–**4**, *a* and *b* as a function of the N–O distance *d*; $\tilde{\nu}$ /cm^{-1}: 1638 (**1**), 1660 (**2**), 1739 (**3**), 1497 (**4**), 1950 (*a*), 1811 (*b*).

The value for **4** was verified by its shift to 1467 cm^{-1} for ^{15}NO. The wavenumbers of the N–O stretches were taken for *a* $[Fe(CN)_5(NO)]^{2-}$ from Ref. [5], for *b* $[Fe(H_2O)_5(NO)]^{2+}$ from Ref. [6]. The reference line is a fit according to Badger's rule applied on the free $NO^{+/0/-}$ species: $\tilde{\nu}$ /cm^{-1} = 856.3 × (d/Å − 0.558)$^{-3/2}$. Calculated N–O distances were used for a better inclusion of $\{FeNO\}^7$ compounds. As explicated in Ref. [6], experimental N–O distances often appear too short due to the slightly tilted NO-group's precession about the Fe–N axis (note the large ellopsoid of the O-atom in Figure 6). For the other species, *d*(exp) and *d*(calc) coincide within narrow limits. Structure optimisation and numerical frequency analyses were performed on the B97-D3-ZORA/def2-TZVP+CPCM(∞) level of theory for the metal-containing species, and in a CASSCF(all valence electrons, all valence orbitals) approach for the free $NO^{-/0/+}$ species, i.e. CASSCF(10/11/12,8).

Right: force constants of the N–O bonds were extracted from the Hessian of the same calculation (using the orca_vib routine of Orca 4.2.1) as above as a function of the QTAIM charges of **1**–**4**, **1'**, *a*, *b*; the line is a fit for the values of the free $NO^{+/0/-}$ species by means of a combined Badger-Gordy approach of the form $f = a\ (q + b)^{3/2}$ with a = 3.292 and b = 2.844. Badger's formula is referenced in the main text, for Gordy's fit see Ref. [7]. The QTAIM charges were calculated by means of Multiwfn, version 3.6.[8]

The frontier orbitals of the vanadate 4

Figure S3 shows the frontier orbitals of the $[V(NO)(tea)]^-$ species **4**. Note the marked extent of static correlation in terms of the antibond population.

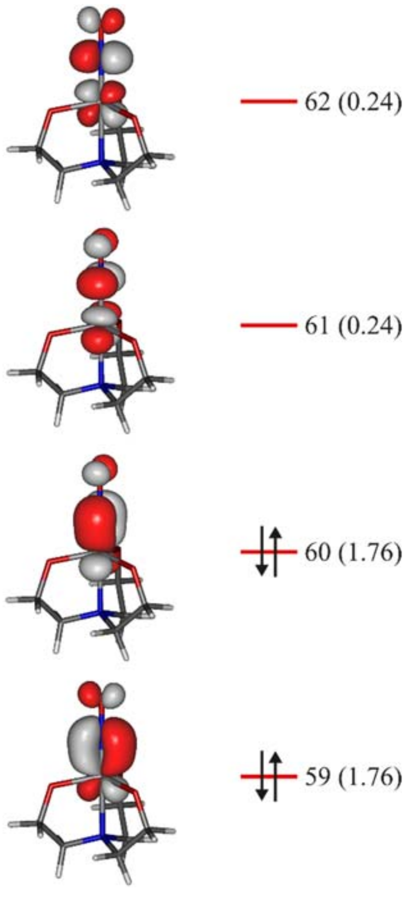

Figure S3. The MOs of the active space of a CASSCF(4,4) calculations, isovalue 0.06 a.u.; the

population is given in parentheses, the arrows represent the ground state's leading 2200 configuration (78 % contribution).

The frontier orbitals of the chromate 2

Figure S4 shows the frontier orbitals of the $[Cr(fpin)_2(NO)]^{2-}$ species **2**. Note the marked extent of static correlation in terms of the antibond population.

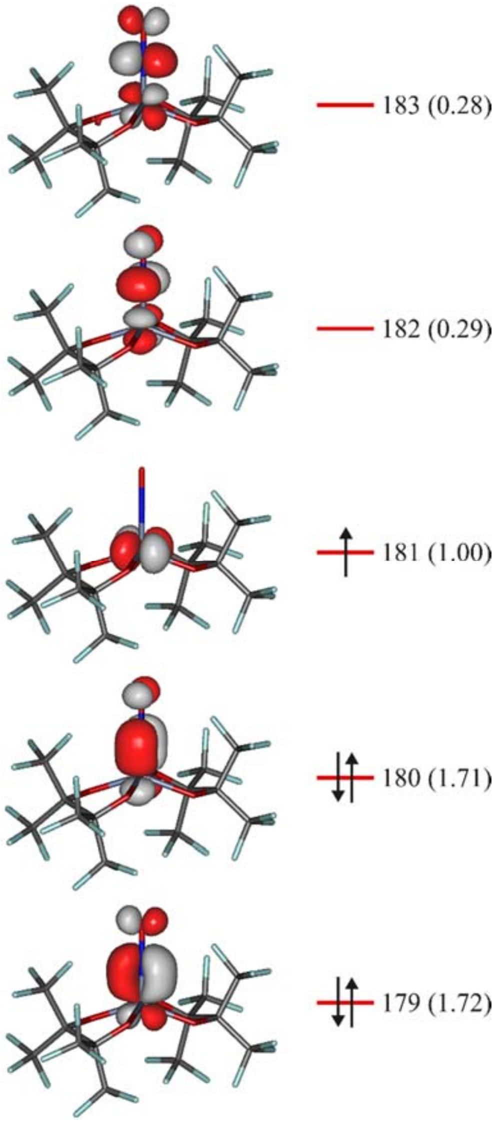

Figure S4. The frontier MOs of a CASSCF(5,7) calculation on **2**, isovalue 0.06 a.u.; the population is given in parentheses, the arrows represent the ground state's leading 2210000 configuration (72 % contribution).

The spin population of the chromate 2

The spin values depend on the method. For the nitrosyl ligand in **2** we found: −0.34 (CASSCF), −0.37 (BP86), −0.54 (B97).

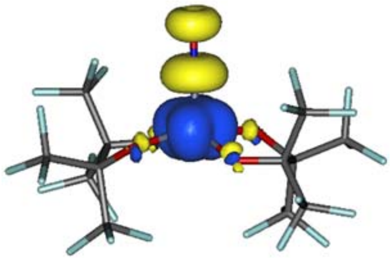

Figure S5. The spin population (isovalue 0.005 a.u.) of the chromate **2** from a B97-D3/def2-TZVP-zora + CPCM(∞) calculation. Blue: α-spin excess, yellow: β-spin excess. Note the spin polarisation along orthogonal interaction of the Cr(d_{xy}) orbital and ligand orbitals: the two Cr–NO π-bonds and the fpin-O–Cr σ-bonds. Spin delocalisation arises from the non-orthogonal Cr(d_{xy})–fpin-O π-interaction.

The frontier orbitals of the ferrate 3

Figure S6 shows the frontier orbitals of the $[Fe(fpin)_2(NO)]^{2-}$ species **3**. Note the marked extent of static correlation in terms of the antibond population.

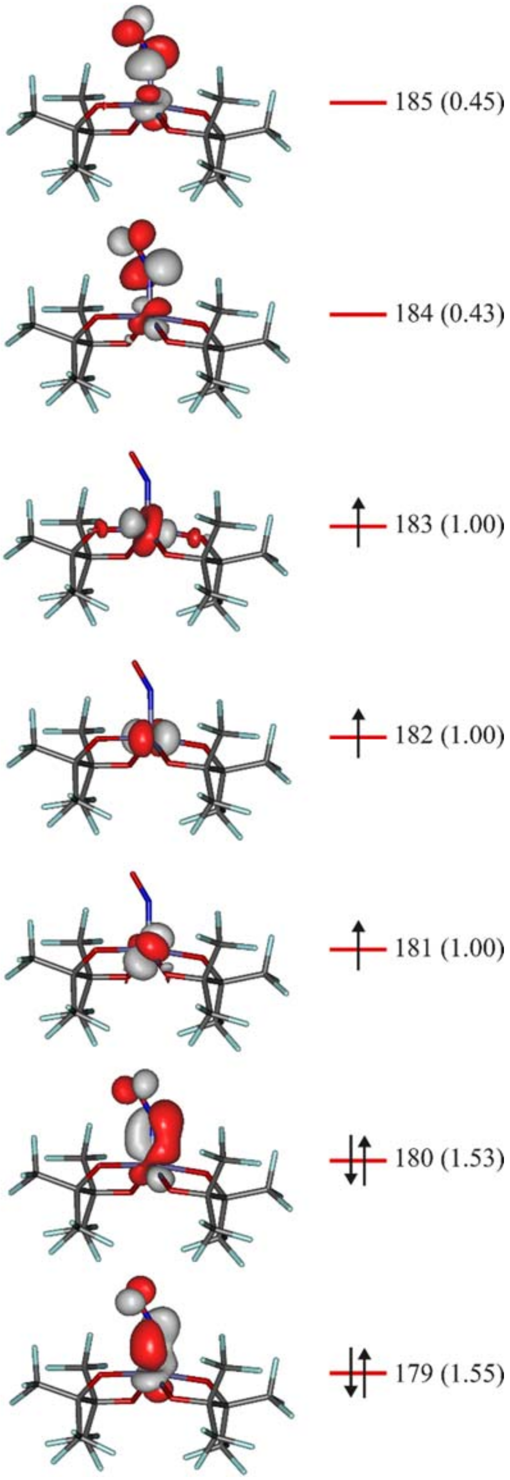

Figure S6. The frontier MOs of a CASSCF(7,7) calculation on **3**, isovalue 0.06 a.u.; the population is given in parentheses, the arrows represent the ground state's leading 2211100 configuration (47 % contribution).

The spin population of the ferrate 3

The spin values depend on the method. For the nitrosyl ligand in **3** we found: −0.87 (CASSCF), −0.94 (BP86), −1.09 (B97).

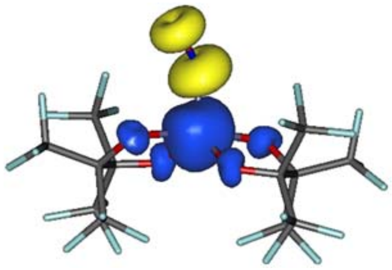

Figure S7. The spin population (isovalue 0.01 a.u.) of the ferrate **3** from a B97-D3/def2-TZVP-zora + CPCM(∞) calculation. Blue: α-spin excess, yellow: β-spin excess. Note the spin polarisation along the two Fe–NO π-bonds. α-Spin delocalisation arises from the central metal to the fpin-O atoms through both σ- and π-interactions.

Details for Scheme 2

As an approximation to the NO-character of a bond the gross population of the diatomic NO fragment was used.

References

[1] S. Kitagawa, M. Munakata, M. Ueda, *Inorg. Chim. Acta* **1989**, *164*, 49-53.

[2] F. Neese, *Wiley Interdisciplinary Reviews: Computational Molecular Science* **2012**, *2*, 73-78.

[3] E. Ramos-Cordoba, V. Postils, P. Salvador, *J. Chem. Theory Comput.* **2015**, *11*, 1501-1508.

[4] E. G. Abucayon, R. L. Khade, D. R. Powell, Y. Zhang, G. B. Richter-Addo, *Angew. Chem. Int. Ed.* **2019**, *58*, 18598-18603.

[5] P. Gütlich, Y. Garcia, T. Woike, *Coord. Chem. Rev.* **2001**, *219–221*, 839-879.

[6] G. Monsch, P. Klüfers, *Angew. Chem. Int. Ed.* **2019**, *58*, 8566-8571.

[7] W. Gordy, *J. Chem. Phys.* **1947**, *15*, 305-310.

[8] T. Lu, F. Chen, *J. Comput. Chem.* **2012**, *33*, 580-592.

Contribution by P. S.

A. Computational details

The wave function and electron densities of all systems have been computed at the BP86-D3(BJ)/def2-tzvp and CASSCF/def2-tzvp including CPCM(water) solvent effects using Gaussian09.[1] In the case of the perfluoro-ligands, the F atoms have been described with the def2-svp basis in the CASSCF calculation due to computational limitations.

The spin-resolved effective fragment orbitals [2], effective oxidation states (EOS) [3] and local spin analysis [4] have been obtained with APOST-3D, [5] The topological fuzzy Voronoi cells (TFVC) atomic definition [6] was used throughout. The first- and second-order reduced density matrices have been obtained with the DMN program. [7]

B. Local spin analysis

Local spins can be extracted from singlet correlated wavefunctions, identifying the presence of *effective* unpaired electrons due to correlation. In this approach, the overall $< S^2 >$ value (zero, in this case) is exactly decomposed into one- and two-center contributions as

$$< \hat{S}^2 > \; = \sum_A < \hat{S}^2 >_{AA} + \sum_{A,B} < \hat{S}^2 >_{AB} .$$

The $< S^2 >_{AA}$ terms account for the presence of local spin in the atom or fragment A, while the $< S^2 >_{AB}$ terms account for the effective spin-spin couplings between the local spins. They are positive if the local spins on A and B are parallel, and negative otherwise. The local spin formulation ensures zero local spins for restricted single-determinant wavefunction, so that the electron pairing in conventional bonds is clearly distinguished from antiferromagnetic interactions, even for a pure singlet without spin density.

In the case of a perfect diradical system, with perfectly localized radical centers on A and B, the expected local spin values would be $< S^2 >_{AA} = < S^2 >_{BB} = ¾$. That is, the $< S^2 >$ value one would obtain for the isolated radical center. Since the overall $< S^2 >$ is zero for a singlet, the diatomic spin contributions that would be obtained are $< S^2 >_{AB} = -3/4$.

In the case of the Co-NO (**1**) and Co-NO (**1′**) species, the local spin analysis obtained from the CASSCF calculations show that only the Co and NO moieties exhibit meaningful local spin. The results obtained can be gathered on 2x2 matrices with the local spin values of Co and NO in the diagonal, and the diatomic spin coupling in the off-diagonal:

Co-NO (**1**) $\begin{pmatrix} 0.276 & -0.266 \\ -0.266 & 0.289 \end{pmatrix}$ Co-NO (**1′**) $\begin{pmatrix} 0.340 & -0.331 \\ -0.331 & 0.349 \end{pmatrix}$

The obtained local spins are significantly below the 0.75 value expected for a diradical. The analysis shows only moderate diradicaloid character of the CASSCF wavefunction, slightly larger for the co-linear **1’** species. Nevertheless, the CASSCF description of the system does contain the partial diradicaloid character that KS-DFT methods detected via two (closed-shell and broken-symmetry) states close in energy.

C. Spin-resolved effective orbitals

Let us consider a system with n orthonormalized occupied molecular orbitals $\varphi_i(\vec{r})$, $i = 1, 2,.., n$ of a given spin case (alpha or beta), and a *fuzzy* division of the 3D-space (atom-in-molecule definition) into N_{at} atomic domains Ω_A defined e.g., by a continuous atomic weight function $w_A(\vec{r})$, fulfilling $w_A(\vec{r}) \geq 0$ and $\sum_A w_A(\vec{r}) \equiv 1$.

Let us for each atom A ($A = 1, 2,..., N_{at}$) form the $n \times n$ Hermitian matrix $\mathbf{Q}^A$ with the elements

$$Q_{ij}^A = \int w_A^*(\vec{r})\varphi_i^*(\vec{r})\varphi_j(\vec{r})w_A(\vec{r})d\vec{r} \tag{1}$$

The matrix $\mathbf{Q}^A$ is essentially the *net* atomic overlap matrix in the basis of the molecular orbitals (MO-s) $\{\varphi_i(\vec{r})\}$. Furthermore, for each atom A we define the *intraatomic* part $\varphi_i^A(\vec{r})$ of every MO as $\varphi_i^A(\vec{r}) \equiv w_A(\vec{r})\varphi_i(\vec{r})$. Thus, $Q_{ij}^A = <\varphi_i^A | \varphi_j^A>$ i.e., $\mathbf{Q}^A$ is the overlap matrix of the orbitals $\{\varphi_i^A(\vec{r})\}$, for every atom A.

We diagonalize the Hermitian matrix $\mathbf{Q}^A$ by the unitary matrix $\mathbf{U}^A$:

$$\boldsymbol{U}^{A+}\boldsymbol{Q}^A\boldsymbol{U}^A = \boldsymbol{\Lambda}^A = diag\ \{\lambda_i^A\}\ . \tag{2}$$

It can be shown that every $\lambda_i^A \geq 0$, as is the case for a proper overlap matrix. For each atom A we obtain n_A ($n_A \leq n$) localized orbitals

$$\chi_\mu(\vec{r}) = \frac{1}{\sqrt{\lambda_\mu^A}}\sum_{i=1}^{n} U_{i\mu}^A \varphi_i(\vec{r}) \qquad \mu = 1, 2,..., n_A, \tag{3}$$

where n_A is the number of non-zero eigenvalues λ_i^A.

The *effective* atomic orbitals $\chi_\mu^A(\vec{r})$ are defined as linear combinations of the *intraatomic* parts $\{\varphi_i^A(\vec{r})\}$ of the MO-s, that is

$$\chi_\mu^A(\vec{r}) \equiv w^A(\vec{r})\chi_\mu(\vec{r}) = \frac{1}{\sqrt{\lambda_\mu^A}}\sum_{i=1}^{n} U_{i\mu}^A \varphi_i^A(\vec{r}) \qquad \mu = 1, 2,..., n_A. \tag{4}$$

The occupation number of each effective atomic orbital (eff-AO) is given by the eigenvalues $0 \leq \lambda_i^A \leq 1$. The sum of the occupation number of the n_A eff-AOs is the *net* population of the atom A for the given spin case:

$$N_{net}^{A} = \sum_{i=1}^{n_A} \lambda_i^A \ . \tag{5}$$

Gross atomic populations associated to each eff-AOs can be derived from the atomic overlap of the localized MO-s (3)

$$\lambda_i^{A,gross} = \int w_A^*(\vec{r})\chi_i^*(\vec{r})\chi_j(\vec{r})d\vec{r} \qquad \text{and } N_{gross}^{A} = \sum_{i=1}^{n_A} \lambda_i^{A,gross} \ . \tag{6}$$

The thus defined gross populations of the eff-AOs add up to the total atomic population derived from the underlying atom-in-molecule method used. It is worth mentioning that in case a disjoint approach is used, such as QTAIM, the eff-AOs net and gross populations are fully equivalent, since in this particular case $w^A(\vec{r}) \equiv w^A(\vec{r})^2 \quad \forall A \wedge \forall \vec{r}$.

The shape and occupation number of the eff-AOs faithfully reproduce the core and valence shells of the atoms; those with occupation numbers close to 1 are associated to core orbitals or lone pairs, whereas those with smaller but significant occupation are identified with the atomic orbitals directly involved in the bonds. The remaining eff-AO-s are marginally occupied and have no chemical significance. For most atoms the number of hybrids with significant occupation number always coincide with the classical minimal basis set, except for those that exhibit hypervalent character.

Notice that the eff-AOs and their occupation numbers can be obtained in the framework of 3D-space analysis even in the absence of an underlying atom-centered basis set, i.e., for plane wave calculations.[5] Another relevant aspect is that the eff-AOs can be easily obtained for any level of theory, provided a first-order density matrix is available (in the case of Kohn-Sham DFT the latter is approximated by the usual Hartree-Fock-like expression). As noted by Mayer,[10] the eff-AOs of a given atom *A* can also be obtained from the diagonalization of the matrix $\mathbf{PS}^A$, where **P** is the LCAO density matrix and $\mathbf{S}^A$ is the intra-atomic overlap matrix in the actual AO basis. This permits the straightforward generalization to correlated wave functions, from which the **P** matrix is usually available.

D. Effective oxidation states (EOS) analysis

The information provided by the eff-AOs and their occupation numbers is used to derive the most appropriate electron configuration of the atoms within the molecule. The integer electrons (alpha and beta, separately) are distributed among the atoms by comparing the occupations of the eff-AOs on different atoms, rather than independently rounding them to the nearest integer. Such strategy also underlines the fact that the OS depends on all atoms of the system and of course on the total number of electrons Moreover, when the number of atoms of the system is large, accidental pseudo degeneracies of the occupation numbers of the eff-AOs are likely to occur, which hinders the assignment of oxidation states. Note that one is usually interested in the oxidation state of the transition metal atoms and the formal charge of their ligands. Hence, a slightly more involved but more efficient strategy is a hierarchical approach, by which molecular

fragments are defined before the eff-AO analysis in a first iteration. That is, instead of eff-AOs we obtain effective *fragment* orbitals (EFOs) by using *fragment weight functions* of the form

$$w_P(\vec{r}) = \sum_{i \in P} w_i(\vec{r}), \tag{7}$$

where the sum runs for all atoms of molecular fragment *P*. The effective oxidation states (EOS) analysis,[3] after molecular fragments have been defined, goes as follows: (i) the alpha eff-AOs that are significantly populated are collected for all fragments, (ii) the eff-AOs are sorted according to decreasing occupation number, and (iii) integer alpha electrons are assigned to the eff-AOs of the fragments with higher occupation number, until the number of alpha electrons is reached. Then, proceed analogously for the beta electrons. By this procedure an effective electronic configuration is obtained for each atom/fragment. The EOS of each atom/fragment is simply given by the difference between its atomic number and the number of alpha and beta electrons that have been assigned to it. This scheme can be safely applied to basis sets including effective core potentials, simply by readily assigning the electrons described by the atomic core potential to the given atom.

The occupation numbers of the *frontier* eff-AO-s, namely the last occupied, λ_{LO}^{σ} and the first unoccupied, λ_{FU}^{σ}, can be used, for each spin case σ, to indicate how close the formal picture given by the EOS is to the actual electronic distribution of the system. Since except for pure ionic systems, significant electron sharing always takes place, these limiting occupation numbers always differ from the ideal one and zero, respectively. When λ_{LO}^{σ} and λ_{FU}^{σ} differ by more than half electron (i.e., a full electron rounding up the difference in occupation number) the assignment of EOS is considered as fully indisputable. For each spin case, a more general reliability index $R^{\sigma}(\%)$ reads

$$R^{\sigma}(\%) = 100\min(1, \max(0, \lambda_{LO}^{\sigma} - \lambda_{FU}^{\sigma} + 1/2)), \tag{8}$$

and then $R(\%) = \min(R^{\alpha}(\%), R^{\beta}(\%))$. That is, the overall *R*(%) index is the minimum value obtained for either the alpha or beta electrons. The larger the *R*(%) value the closer the overall assignment of the EOS is to the actual electronic structure of the system. Note that *R*(%) can take values formally from 0 to 100, where values below 50% indicate that the assignment of the electrons has not followed *an aufbau* principle according to the occupation numbers of the eff-AOs. The latter avenue can be used *to measure to which extent* the molecular system conforms with a given set of oxidation states, rather than which are the most appropriate formal oxidation states.

If the frontier eff-AOs for any spin case are degenerate (same occupation number) and belong to different fragments, a value of *R*=50% would be obtained. In that case, however, one may choose to assign half-electron to each of the two atoms/fragments involved (or, in general, a fraction of the last *m* electrons that must be distributed among n_d degenerate eff-AOs), to accommodate e.g. genuine mixed-valence situations. Then, the λ_{FU}^{σ} value to be used to evaluate $R^{\sigma}(\%)$ is the one immediately below the degenerate λ_{LO}^{σ} value. We use such an approach only when the degeneracies are due to symmetry. Alternatively, one might

define a (small) threshold to consider two or more eff-AO as pseudo-degenerate when their occupation numbers are close enough.[3]

E. EOS analysis and %NO character

The EFOs of all ligands and central metal atom are obtained as described in section B. Visual inspection of the valence EFOs typically evidence a correspondence between the dangling valences resulting from the formal splitting of the bonds required to isolate the given fragment from the rest of the molecule. In the case of the M-NO interaction, one can identify a pair of hybrid π^* EFOs of the NO moiety (occupation numbers gathered in Table S1) with their lobes matching to d-type hybrids on M. The set of corresponding EFOs and their occupations are displayed in Figures S1-S10. In some cases, (in particular species **4** and beta channel for **b**) a fully complementarity of the EFO lobes is not observed. In that case, the frontier EFOs of the metal are used to estimate the %NO character.

The percentage of NO character of the M-NO bonds can be estimated by different approaches, e.g. NBO analysis or any population analysis of the corresponding localized orbitals.[11] In this work, we estimate the %NO of each M-NO bonding interaction and spin case from the occupations of the EFOs shown in the Figures S8-S17 as

$$\%NO_i^\sigma = 100 \frac{\lambda_{i,\pi-NO}^\sigma}{\lambda_{i,\pi-NO}^\sigma + \lambda_{i,d-M}^\sigma}, \tag{9}$$

where $\lambda_{i,\pi-NO}^\sigma$ and $\lambda_{i,d-M}^\sigma$ are the occupations of the ith pair of π^* and d-hybrid of spin σ, respectively. The results for all species considered in this work are gathered on Table S2, and graphically sketched on Scheme 2 of the manuscript.

TABLE S1. Gross occupations of the π* effective fragment orbitals (EFOs) of NO for the alpha and beta spin channels (total occupation in bold) and %NO character. OS assignation for the NO ligand and overall reliability index (R%). EFO occupations in red are considered unoccupied upon EOS analysis.

Species	M-N-O angle	Charge NO (TFVC)	OS	R(%)	π*	%NO	π *	%NO
$\{CoNO\}^8$					0.443	35.0	0.247	31.3
**1**	120.6	-0.137	+1	58.4	0.443	35.0	0.247	31.3
					**0.885**		**0.494**	
$\{CoNO\}^8$					0.471	33.7	0.244	32.5
**1´**	180.0	-0.219	+1	53.1	0.471	33.7	0.244	32.5
					**0.942**		**0.488**	
$[Fe(CN)_5(NO)]^{2-}$					0.304	32.0	0.300	31.6
a	176.3	-0.073	+1	83.3	0.304	32.0	0.300	31.6
					**0.608**		**0.600**	
$\{VNO\}^4$					0.495	52.2	0.495	52.2
**4**	177.9	-0.856	-3	55.0	0.495	52.2	0.495	52.2
					**0.991**		**0.990**	
$\{CrNO\}^5$					0.388	38.7	0.362	37.9
**2**	179.5	-0.657	-1	62.8	0.546	57.4	0.528	57.1
					**0.934**		**0.890**	
$\{FeNO\}^7$					0.210	20.4	0.206	18.1
**3**	168.5	-0.500	-1	81.2	0.621	68.0	0.616	67.0
					**0.831**		**0.822**	
$[Fe(H_2O)_5(NO)]^{2+}$					0.165	15.0	0.150	13.9
b	162.3	-0.043	+1	54.4	0.435	47.3	0.417	45.5
					**0.600**		**0.567**	

CASSCF(7,12) results, as described in [8] for species ***b***. CASSCF(6,6) results for species ***a***

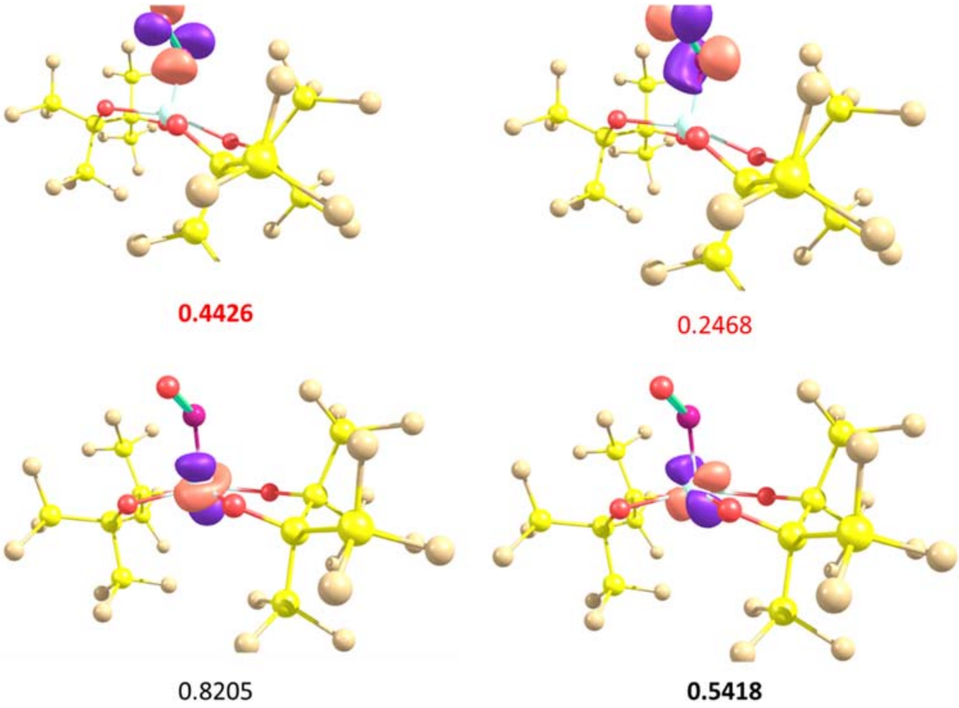

Figure S8: EFO of the NO π* hybrid orbitals and d-type hybrids on the central metal atom for species **1**. EFO gross occupations in red are considered unoccupied upon EOS analysis. Last occupied and first unoccupied EFOs marked in bold.

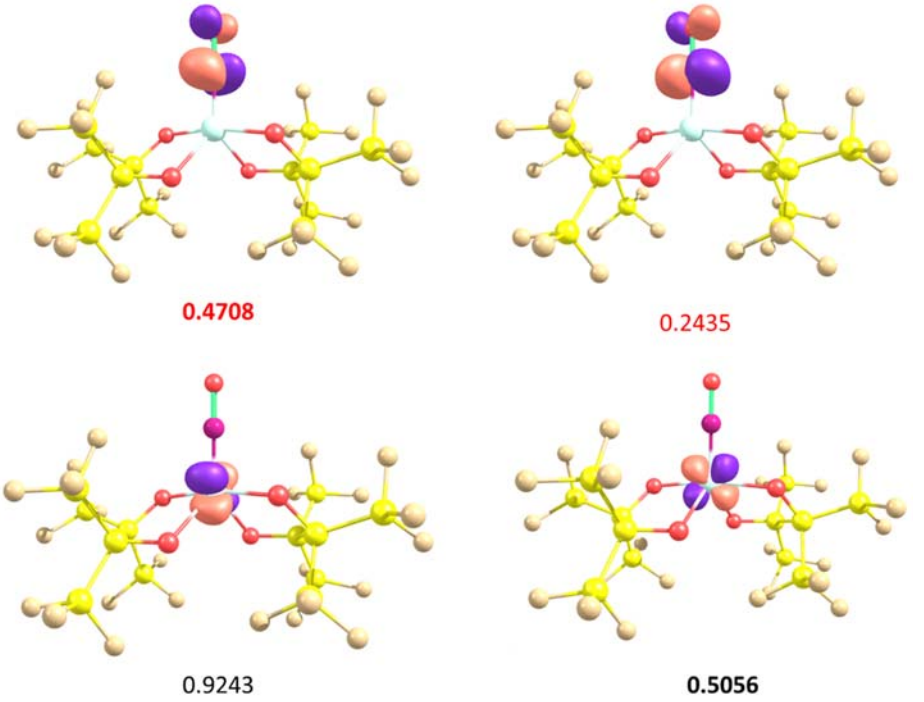

Figure S9: EFO of the NO π* hybrid orbitals and d-type hybrids on the central metal atom for species **1'**. EFO gross occupations in red are considered unoccupied upon EOS analysis. Last occupied and first unoccupied EFOs marked in bold.

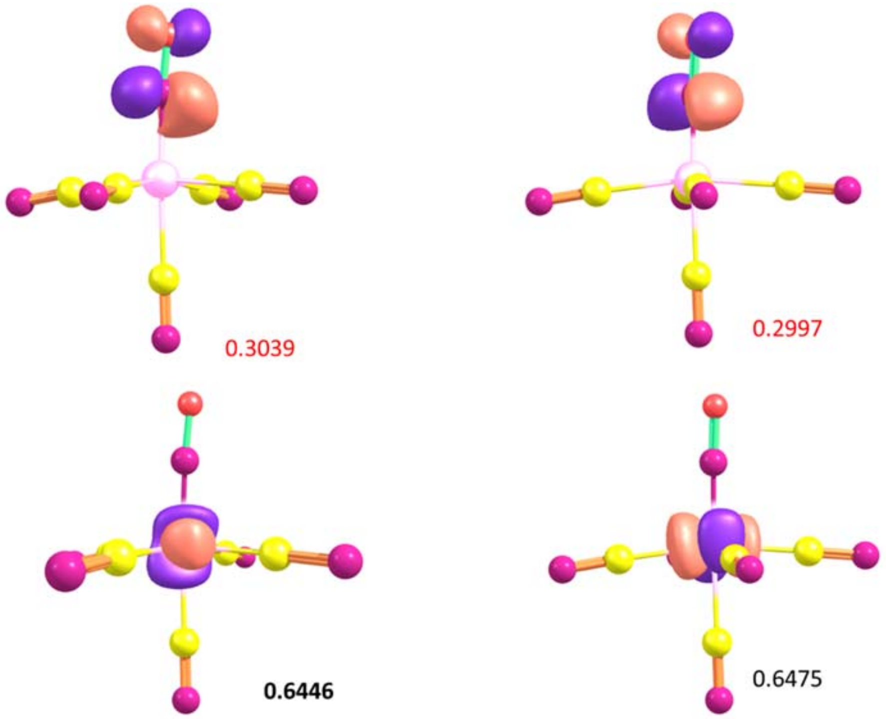

Figure S10: EFO of the NO π^* hybrid orbitals and d-type hybrids on the central metal atom for species **a**. EFO gross occupations in red are considered unoccupied upon EOS analysis. Last occupied and first unoccupied EFOs marked in bold.

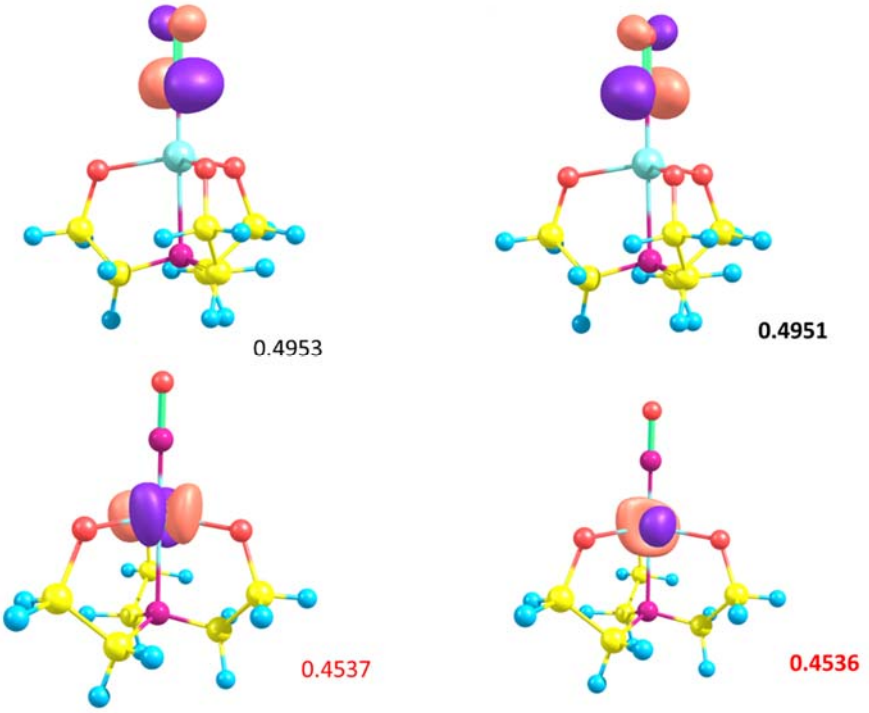

Figure S11: EFO of the NO π^* hybrid orbitals and d-type hybrids on the central metal atom for species **4**. EFO gross occupations in red are considered unoccupied upon EOS analysis. Last occupied and first unoccupied EFOs marked in bold.

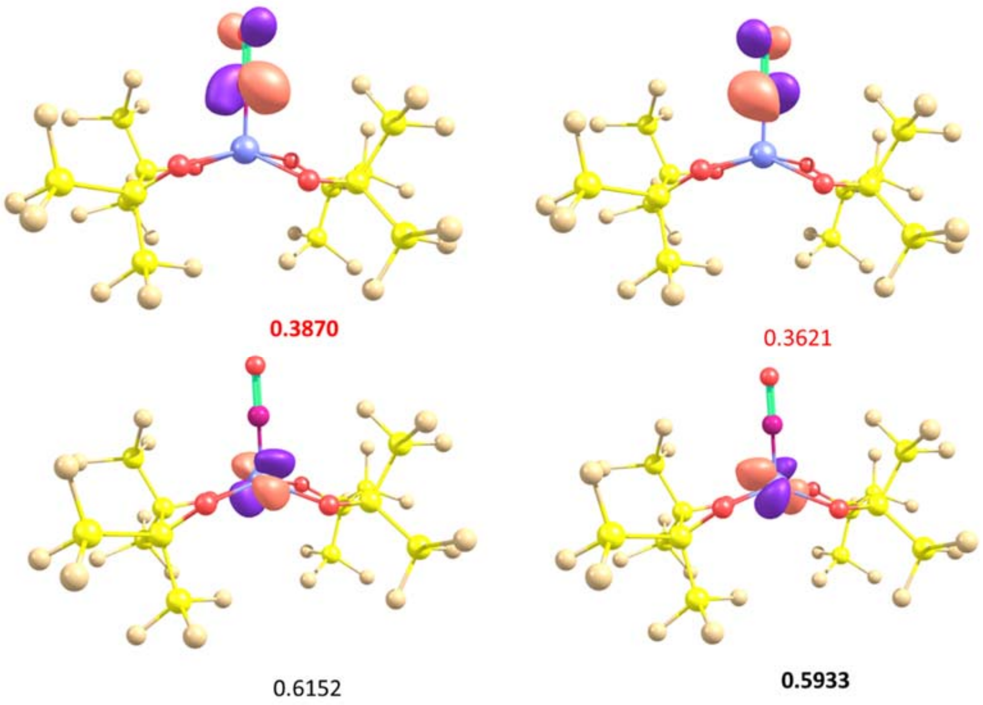

Figure S12: Alpha EFO of the NO π^* hybrid orbitals and d-type hybrids on the central metal atom for species **2**. EFO gross occupations in red are considered unoccupied upon EOS analysis. Last occupied and first unoccupied EFOs marked in bold.

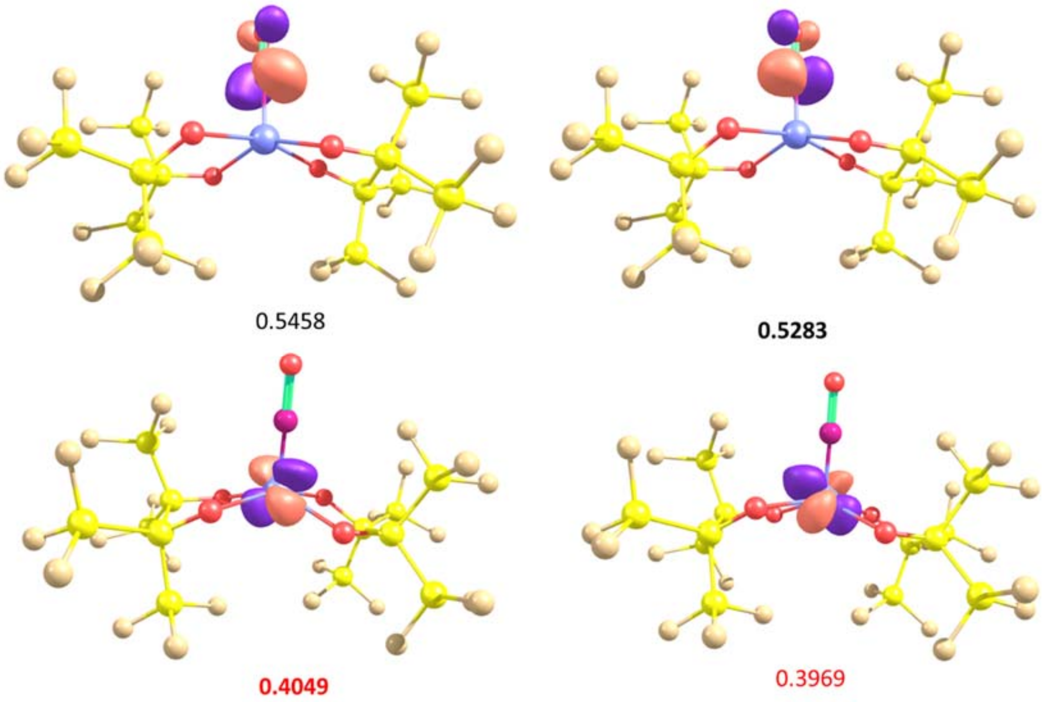

Figure S13: Beta EFO of the NO π^* hybrid orbitals and d-type hybrids on the central metal atom for species **2**. EFO gross occupations in red are considered unoccupied upon EOS analysis. Last occupied and first unoccupied EFOs marked in bold.

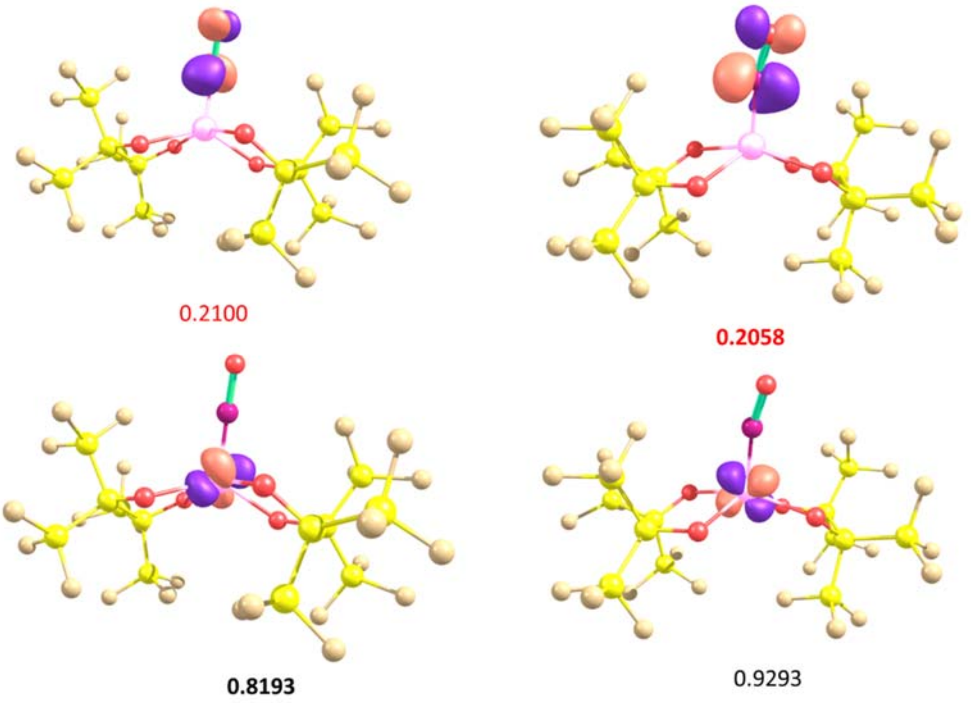

Figure S14: Alpha EFO of the NO π^* hybrid orbitals and d-type hybrids on the central metal atom for species **3**. EFO gross occupations in red are considered unoccupied upon EOS analysis. Last occupied and first unoccupied EFOs marked in bold.

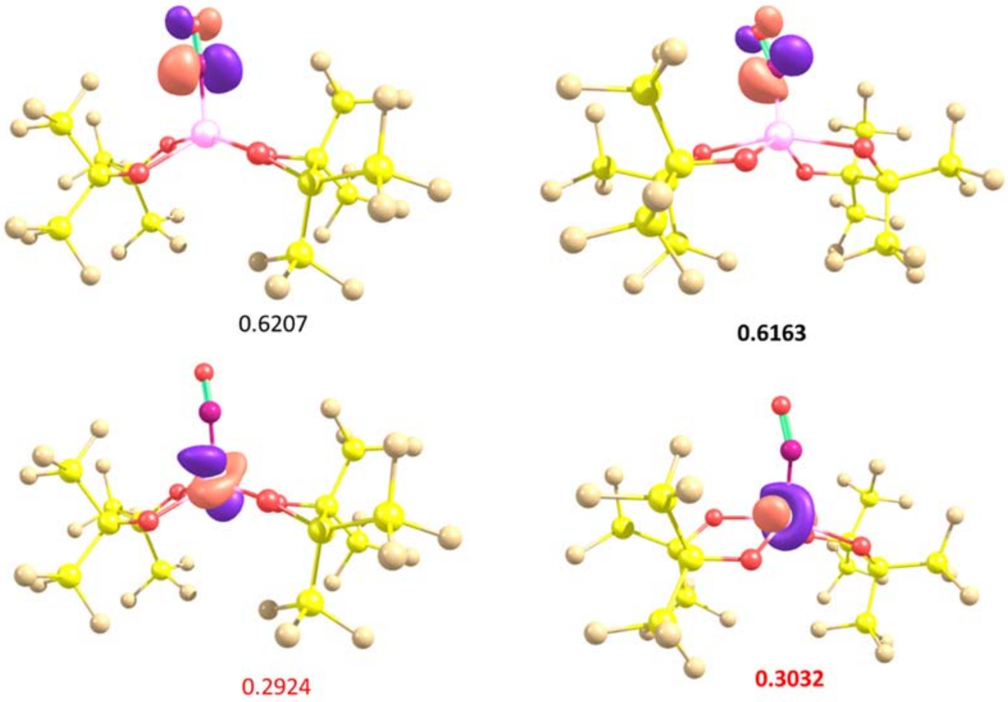

Figure S15: Beta EFO of the NO π^* hybrid orbitals and d-type hybrids on the central metal atom for species **3**. EFO gross occupations in red are considered unoccupied upon EOS analysis. Last occupied and first unoccupied EFOs marked in bold.

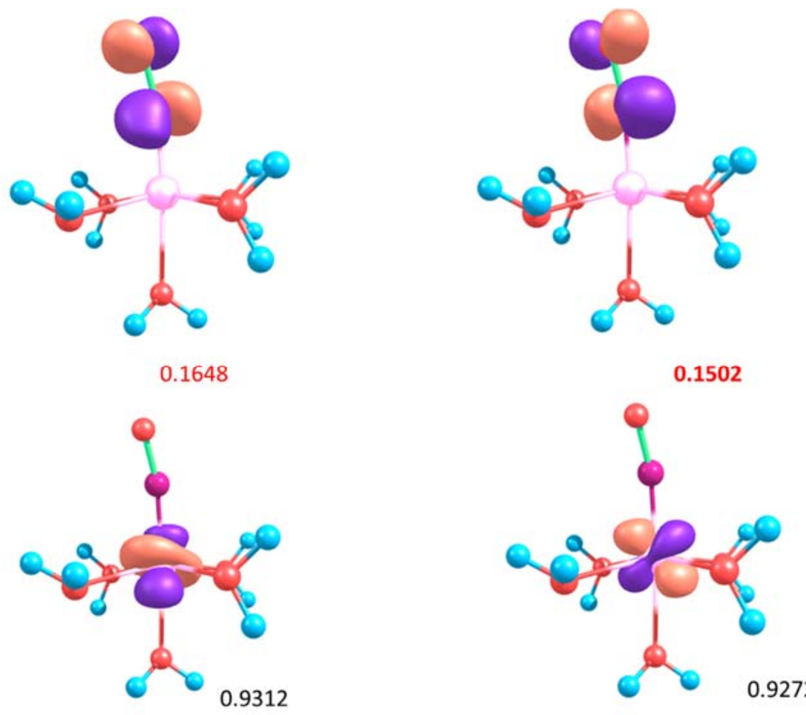

Figure S16: Alpha EFO of the NO π* hybrid orbitals and d-type hybrids on the central metal atom for species **b**. EFO gross occupations in red are considered unoccupied upon EOS analysis. Last occupied and first unoccupied EFOs marked in bold.

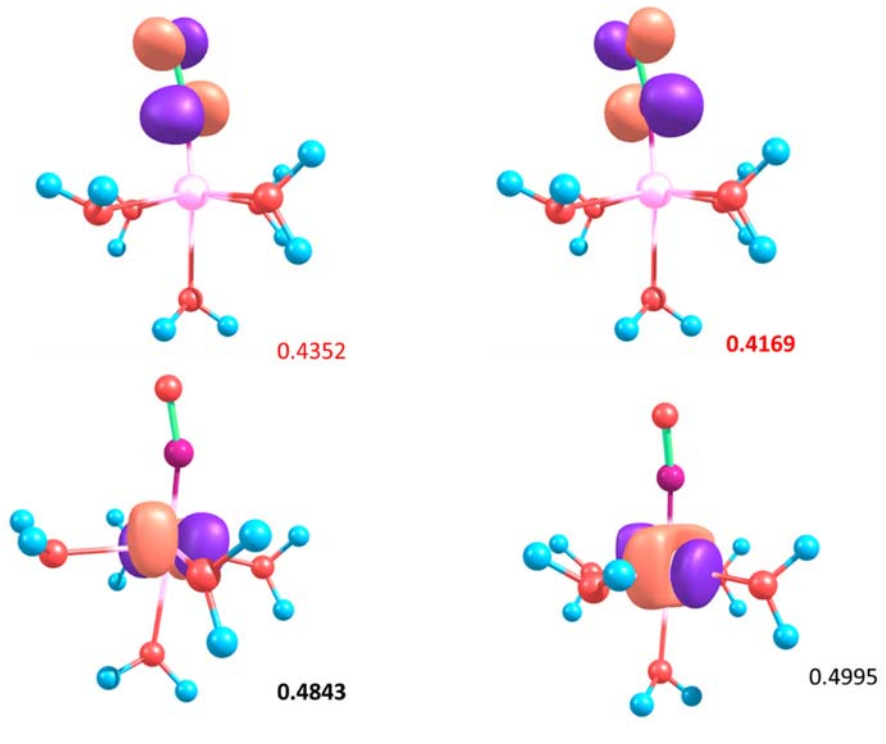

Figure S17: Beta EFO of the NO π* hybrid orbitals and d-type hybrids on the central metal atom for species **b**. EFO gross occupations in red are considered unoccupied upon EOS analysis. Last occupied and first unoccupied EFOs marked in bold.

References

[1] Gaussian 16, Revision A.03, M. J. Frisch, G. W. Trucks, H. B. Schlegel, G. E. Scuseria, M. A. Robb, J. R. Cheeseman, G. Scalmani, V. Barone, B. Mennucci, G. A. Petersson, H. Nakatsuji, M. Caricato, X. Li, H. P. Hratchian, A. F. Izmaylov, J. Bloino, G. Zheng, J. L. Sonnenberg, M. Hada, M. Ehara, K. Toyota, R. Fukuda, J. Hasegawa, M. Ishida, T. Nakajima, Y. Honda, O. Kitao, H. Nakai, T. Vreven, J. J. A. Montgomery, J. E. Peralta, F. Ogliaro, M. Bearpark, J. J. Heyd, E. Brothers, K. N. Kudin, V. N. Staroverov, R. Kobayashi, J. Normand, K. Raghavachari, A. Rendell, J. C. Burant, S. S. Iyengar, J. Tomasi, M. Cossi, N. Rega, J. M. Millam, M. Klene, J. E. Knox, J. B. Cross, V. Bakken, C. Adamo, J. Jaramillo, R. Gomperts, R. E. Stratmann, O. Yazyev, A. J. Austin, R. Cammi, C. Pomelli, J. W. Ochterski, R. L. Martin, K. Morokuma, V. G. Zakrzewski, G. A. Voth, P. Salvador, J. J. Dannenberg, S. Dapprich, A. D. Daniels, O. Farkas, J. B. Foresman, J. V. Ortiz, J. Cioslowski and D. J. Fox, Journal, 2016.Gaussian, Inc., Wallingford CT, 2016.

[2] I. Mayer, P. Salvador, *J. Chem. Phys.* **2009***,* 130**,** 234106-6.

[3] E. Ramos-Cordoba, V. Postils, P. Salvador, J. Chem. Theory Comput. **2015**, 11, 1501-1508.

[4] E. Ramos-Cordoba, E. Matito, I. Mayer, P. Salvador, J. Chem. Theory Comput. **2012**, 8, 1270 –1279.

[5] P. Salvador, E. Ramos-Cordoba, APOST-3D program, Universitat de Girona: Girona, Spain, 2012.

[6] P. Salvador, E. Ramos-Cordoba, *J. Chem. Phys.* **2013**, 139, 071103-4.

[7] E. Matito and F. Feixas, DMn program, University of Girona (Spain) and University of Szczecin (Poland), 2009

[8] G. Monsch, P. Klüfers, Angew. Chem. Int. Ed. **2019**, 58, 8566-8571.

[9] I. Bakó, A. Stirling, A.P. Seitsonen, I. Mayer, *Chem. Phys. Lett.* **2013**, 563, 97-101.

[10] I. Mayer, *Can. J. Chem.* **1996**, 74, 939−942.

[11] I. G. Pallares, T. C. Brunold, Inorg. Chem. 2014, 53, 7676-7691.
